# 转录因子EB及其靶基因在硼替佐米治疗多发性骨髓瘤中的作用及分子机制

**DOI:** 10.3760/cma.j.cn121090-20250614-00276

**Published:** 2025-11

**Authors:** 荣娟 张, 紫琳 王, 小敏 石, 姝苑 张, 玮 王, 铭帅 马, 冲 栗, 翠红 谷, 志华 张

**Affiliations:** 1 承德医学院附属医院血液内科，承德 067000 Department of Hematology, The Affiliated Hospital of Chengde Medical College, Chengde 067000, China; 2 河北省泛血管疾病重点实验室，承德 067000 Hebei Key Laboratory of Panvascular Diseases, Chengde 067000, China

**Keywords:** 转录因子, 多发性骨髓瘤, 硼替佐米, 基因表达调控, 自噬, Transcription factors, Multiple myeloma, Bortezomib, Gene expression regulation, Autophagy

## Abstract

**目的:**

探索转录因子EB（TFEB）及其靶基因在硼替佐米治疗多发性骨髓瘤（MM）中的作用及分子机制。

**方法:**

通过GTRD网站（http://gtrd.biouml.org/）预测TFEB靶基因，筛选出Ptch1基因。实时荧光定量PCR和Western blot法检测硼替佐米作用于MM细胞系RPMI8226和U266后Ptch1的表达变化。siRNA-TFEB分别转染RPMI8226和U266细胞系，实时荧光定量PCR和Western blot法检测Ptch1/Hedgehog信号通路关键因子（Ptch1、Gli1）的mRNA和蛋白相对表达情况。在MM细胞系中用慢病毒转染过表达Ptch1，吖啶橙染色观察自噬现象，Western blot法检测各组细胞自噬相关因子（LC3B、Beclin-1、Lamp-1）蛋白相对表达量，溶酶体荧光探针观察溶酶体数量变化。

**结果:**

硼替佐米（6.0×10^−6^ mmol/L）处理RPMI8226和U266细胞系24 h，硼替佐米组Ptch1的mRNA和蛋白的相对表达水平均较空白对照组降低（*P*值均<0.05），转染siRNA-TFEB后逆转了硼替佐米对Hedgehog通路中关键因子Ptch1、Gli的抑制作用。Ptch1过表达显著减少了经硼替佐米处理的RPMI8226和U266细胞系中自噬相关因子LC3B、Beclin1、Lamp1蛋白的相对表达量（*P*值均为0.001）。吖啶橙染色显示两种细胞系酸性囊泡数量均减少（*P*值均为0.001），反映溶酶体数量的溶酶体探针相对荧光表达亦减少（RPMI8226和U266细胞系的*P*值分别为0.001、0.007）。

**结论:**

敲低TFEB可靶向促进Ptch1/Hedgehog信号通路表达而减少硼替佐米诱导的MM细胞自噬，并逆转硼替佐米对MM细胞系的增殖抑制作用。

## Abstract (en)

**Objective:**

To investigate the role and molecular mechanisms of transcription factor EB（TFEB）and its target genes in the treatment of multiple myeloma（MM）with bortezomib.

**Methods:**

TFEB target genes were predicted using the GTRD database（http://gtrd.biouml.org/）, identifying Ptch1 gene for further study. Expression changes of Ptch1 in RPMI8226 and U266 MM cell lines after bortezomib treatment were assessed by real time fluorogenic quantitative PCR（RT-qPCR）and Western blot. RPMI8226 and U266 cell lines were transfected with siRNA-TFEB, and mRNA and protein levels of key factors（Ptch1, Gli1）in the Ptch1/Hedgehog signaling pathway were measured by RT-qPCR and Western blot. Furthermore, Ptch1 was overexpressed in MM cell lines via lentiviral transduction. Autophagy was evaluated by acridine orange staining, and protein levels of LC3B, Beclin-1, and Lamp-1 were measured by Western blot. Lysosomal quantity changes were assessed by lysosomal fluorescent probes.

**Results:**

Bortezomib（6.0×10^−6^ mmol/L, 24 h）significantly reduced Ptch1 mRNA and protein levels in both cell lines compared with blank control group（all *P*<0.05）. siRNA-TFEB transfection reversed bortezomib's inhibition of Hedgehog pathway key factors Ptch1 and Gli. Ptch1 overexpression in bortezomib-treated RPMI8226 and U266 cells significantly reduced the relative expression of autophagy-related proteins LC3B, Beclin-1, and Lamp-1（all *P*＝0.001）. Acridine orange staining showed fewer acidic vesicular organelles within two cell lines（all *P*＝0.001）. The relative fluorescence expressions of lysosomal probes reflecting the number of lysosomes were also decreased（*P* values of RPMI8226 and U266 cell lines were 0.001 and 0.007, respectively）.

**Conclusion:**

The knockdown of TFEB can specifically promote the expression of the Ptch1/Hedgehog signaling pathway, thereby reducing bortezomib-induced autophagy in MM cells and reversing the inhibitory effect of bortezomib on the proliferation of MM cell lines.

多发性骨髓瘤（MM）是常见的血液系统恶性肿瘤，其发病率和死亡率均呈现逐年上升趋势[1]。尽管近年来MM的药物和造血干细胞移植治疗取得了一定进展，但MM仍是一种难以治愈的疾病，患者生存期较短，预后较差。因此，深入探讨MM的发病机制，寻找新的治疗靶点，已成为当前血液学研究领域的热点和难点。转录因子EB（TFEB）是溶酶体自噬途径中重要的转录调节因子。TFEB作为MiTF/TFE家族中的一员，在进化中高度保守，在多种细胞中均表达[2]–[7]，可根据机体的需要控制其靶基因产物的数量[8]–[9]。本课题组前期一系列研究提示，TFEB介导的自噬和溶酶体机制参与了硼替佐米治疗MM[10]。由于自噬调控机制的复杂性与多样性，本研究拟结合生物信息学分析，探讨TFEB及其靶基因在硼替佐米治疗MM中的作用及其分子机制。

## 材料与方法

1. 材料和试剂：RPMI8226及U266细胞购自上海中美合资博慧斯生物医药科技有限公司。硼替佐米（规格5 mg/瓶，型号S1013）购自美国Selleck公司，兔抗人LC3B、Beclin-1、LAMP1、TFEB、Gli1单克隆抗体、Ptch1多克隆抗体购自英国Abcam公司，β-actin单克隆抗体为美国Santa Cruz Biotechnlogy公司产品，羊抗兔二抗购自美国KPL公司，TRIzol试剂购自美国Invitrogen公司，反转录试剂盒及引物均为美国Invitrogen公司产品。溶酶体红色荧光探针（Lyso-Tracker Red）购自上海碧云天生物技术有限公司，LipofectamineTM 3000 Transfection Reagent为美国Invitrogen公司产品，Ptch1过表达慢病毒购自上海吉凯基因科技有限公司，实时定量PCR仪为美国Invitrogen公司产品，DYY-6C电泳仪为北京六一生物科技有限公司产品。

2. 细胞培养：RPMI8226及U266细胞培养于含青霉素（100 U/m1）、链霉素（100 g/m1）的10％胎牛血清RPMI 1640培养基中，置于饱和湿度、37 °C、5％ CO_2_培养箱中常规传代培养，取对数生长期细胞用于后续实验。

3. 构建敲低TFEB细胞系以及实验分组：采用小干扰RNA（siRNA）在MM细胞系中敲低TFEB的表达。从生长状况良好的RPMI8226和U266细胞中取样，准备转染试剂溶液1（5 µl Lipofectamine 3000+125 µl培养基混匀，静置5 min）和溶液2（2×10^−7^ mmol siRNA +125 µl培养基混匀，静置5 min），溶液1和溶液2混匀逐滴加入各组细胞，摇动培养板混匀，于37 °C，5％CO_2_培养箱中培养24～48 h，采用实时荧光定量PCR（RT-qPCR）和Western blot技术检测转染细胞中TFEB的表达水平。实验分为空载体（NC siRNA）组、硼替佐米作用于空载体（Bor+NC siRNA）组、转染siRNA-TFEB（si-TFEB）组、硼替佐米作用于转染siRNA-TFEB（Bor + si-TFEB）组。根据既往实验结果，选取能保持细胞存活率且可引起自噬表达变化的硼替佐米浓度（6.0×10^−6^ mmol/L）作用24 h进行后续实验[11]。

4. 硼替佐米作用于MM RPMI8226细胞系后差异表达基因分析及预测靶基因：收取硼替佐米作用后的RPMI8226细胞（Bor组）及空白对照组（Con组）细胞，抽提RNA后进行高通量测序分析，每个处理组设置3个复孔。采用DNBSEQ平台检测Con组和Bor组差异mRNA的基因表达。使用差异表达测序分析（DESeq2，v1.4.5）中的负二项分布模型进行差异基因检测，条件为*Q*值≤0.05或错误发现率≤0.001。通过GTRD网站（http://gtrd.biouml.org/）预测TFEB靶基因。

5. CCK-8法检测各组细胞增殖情况：取对数生长期细胞接种于96孔板内，每孔8×10^3^个细胞，每组设计5个复孔，每孔加入10 µl CCK-8溶液（避免产生气泡），将96孔板置于37 °C培养箱中孵育2 h，置于酶标仪中测定其在450 nm处的吸光度（*A*_450_）值，计算细胞存活率：细胞存活率（％）=（*A*_实验组_−*A*_空白组_）/（*A*_对照组_−*A*_空白组_）×100％。

6. RT-qPCR：使用TRIzol试剂提取总RNA，反转录试剂盒合成cDNA。采用SuperReal荧光定量（SYBR Green）进行RT-qPCR检测。所有结果均用GAPDH进行标准化处理。使用2^−△△Ct^法，通过目标基因与内参基因Ct值的差异计算基因表达变化。

7. Western blot：使用RIPA裂解液提取细胞中总蛋白，使用BCA试剂盒测定蛋白浓度。取等量蛋白样品上样，进行SDS-PAGE电泳、转膜，5％脱脂奶粉封闭2 h。4 °C条件下LC3Ⅱ（稀释浓度为1∶5 000）、Beclin-1（稀释浓度为1∶5 000）、Lamp1（稀释浓度为1∶5 000）、Ptch1（稀释浓度为1∶2 000）、Gli1（稀释浓度为1∶2 000）一抗摇床过夜，在室温下孵育二抗（稀释浓度为1∶10 000）2 h，暗室曝光。采用成像系统进行蛋白表达检测，通过ImageJ进行灰度值计算。

8. 吖啶橙染色荧光显微镜下观察细胞自噬现象：取对数生长期细胞，以8×10^4^个/ml的密度接种于6孔板。按上述分组条件孵育相应时间后，离心弃培养液，加入2 mg/ml吖啶橙室温避光孵育15 min，PBS清洗涂片后，置于荧光显微镜下观察、摄片。

9. Lyso-Tracker Red检测各组细胞溶酶体数量变化：实验分组同吖啶橙染色。取1 µl Lyso-Tracker Red加入15 ml细胞培养液中混匀，37 °C避光孵育30 min即得到Lyso-Tracker Red工作液。在各组细胞中加入工作液，37 °C避光孵育30 min，离心，PBS洗涤细胞2次，加入Hoechst染色液避光孵育30 min染核，PBS洗涤细胞2次，将细胞涂于载玻片后，盖上盖玻片，置于荧光显微镜下观察。

10. Ptch1细胞转染与后续实验分组：转染Ptch1对照质粒单克隆细胞（OE-NC）组转染Ptch1对照质粒；转染Ptch1过表达质粒单克隆细胞（OE-Ptch1）组转染Ptch1过表达质粒。分别将OE-NC和OE-Ptch1慢病毒质粒按感染复数（MOI）100∶1感染对数生长期的RPMI8226和U266细胞，约72 h后观察各组感染效率。将各组细胞继续培养于含适当浓度嘌呤霉素的培养液中筛选48 h，空细胞+嘌呤霉素组全部死亡，感染病毒组存活下来的是阳性细胞，对阳性细胞行混合克隆稳定株及单克隆稳定株筛选，收集细胞进行RT-qPCR和Western blot鉴定，选择鉴定结果正常的单克隆细胞用于后续试验。后续实验分组为OE-NC组、OE-Ptch1组、OE-NC+Bor组（硼替佐米作用于OE-NC组）、OE-Ptch1+Bor组（硼替佐米作用于OE-Ptch1组）。

11. 统计学处理：采用SPSS 23.0软件对数据进行统计分析，数据用*x*±*s*表示。多样本均数的比较采用单因素方差分析，组间比较采用最小显著差异法*t*检验，*P*<0.05表示差异有统计学意义。

## 结果

1. 硼替佐米作用于MM RPMI8226细胞系后差异表达基因分析：DNBSEQ平台检测组间差异mRNA，结果显示，Con组和Bor组有差异mRNA基因4 153个，其中1 238个表达上调，2 915个表达下调。

GTRD网站分析TFEB的靶基因共有1 620个，Hedgehog（Hh）信号通路相关因子共18个，包括SHH、IHH、DHH、Ptch1、Ptch2、SMO、GLI1、GLI2、GLI3、SUFU、KIF7、HIP、Cyclin D1、Myc、Nanog、Oct4、Bcl-2、Survivin。TFEB靶基因预测、差异mRNA、Hh信号通路的共同因子为Ptch1，待后续实验验证。

2. RT-qPCR和Western blot检测Ptch1表达：硼替佐米（6.0×10^−6^ mmol/L）处理RPMI8226和U266细胞系24 h，RT-qPCR结果显示，与Con组相比，Bor组Ptch1在两个细胞系中的mRNA相对表达量分别为0.44±0.07、0.52±0.02；Western blot结果显示，与Con组相比，Bor组Ptch1的蛋白相对表达水平分别为0.58±0.03、0.78±0.04，均低于正常对照组（*P*值均<0.05），与测序数据中基因的表达变化趋势一致（图1）。

**图1:**
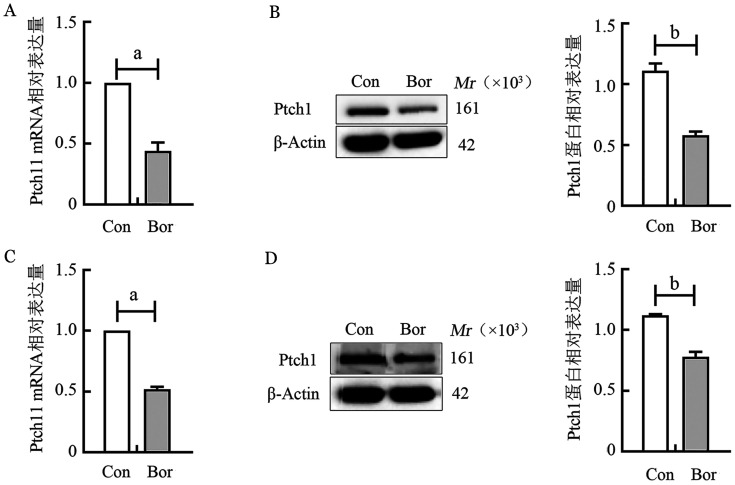
实时荧光定量PCR和Western blot法检测硼替佐米作用于RPM I8226细胞系（A、B）和U266细胞系（C、D）后Ptch1表达变化 **注** ^a^*P*<0.001，^b^*P*<0.01；Con：对照组；Bor：硼替佐米组

3. TFEB对MM细胞增殖及Ptch1/Hh表达的影响：在RPMI8226细胞系中，NC siRNA、Bor+NC siRNA组细胞存活率分别为（100±0）％、（36.73±1.78）％；在U266细胞系中，NC siRNA、Bor+NC siRNA组细胞存活率分别为（100±0）％、（30.81±5.99）％；提示硼替佐米对MM细胞系RPMI8226和U266具有明显的增殖抑制作用（*P*值均<0.05）。将硼替佐米作用于转染si-TFEB的MM细胞系后，MM细胞系RPMI8226和U266的细胞存活率分别为（44.29±1.14）％、（43.81±4.74）％，si-TFEB减弱了硼替佐米对MM细胞系的增殖抑制作用（*P*值均<0.05）（图2）。

**图2:**
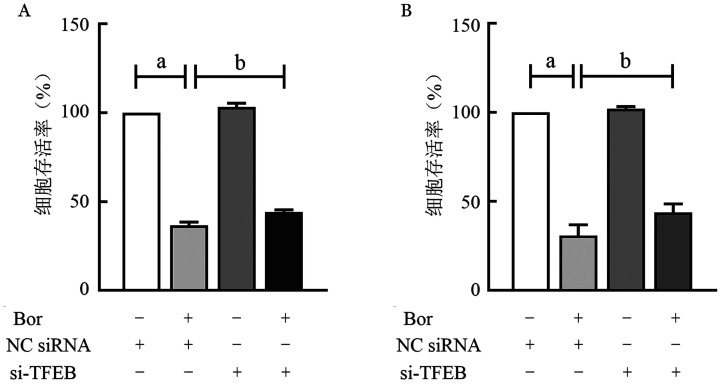
转染si-TFEB对RPMI 8226细胞系（A）和U266细胞系（B）细胞增殖的影响 **注** ^a^*P*<0.001，^b^*P*<0.01；Bor：硼替佐米；NC siRNA：空载体；si-TFEB：小干扰RNA-转录因子EB

在RPMI8226细胞系中，NC siRNA、si-TFEB、Bor+NC siRNA、Bor+si-TFEB四组细胞Ptch1的mRNA相对表达量分别为1.00±0、1.50±0.15、0.41±0.05、0.80±0.05，蛋白相对表达量分别为0.49±0.05、0.46±0.05、0.21±0.02、0.46±0.03；Gli的mRNA相对表达量分别为1.00±0、1.39±0.12、0.25±0.01、0.60±0.03，蛋白相对表达量分别为1.01±0.02、1.33±0.29、0.62±0.01、0.73±0.01（图3）。在U266细胞系中，NC siRNA、si-TFEB、Bor+NC siRNA、Bor+si-TFEB四组细胞Ptch1的mRNA相对表达量分别为1.00±0、1.35±0.02、0.42±0.01、0.78±0.06，蛋白相对表达量分别为0.44±0.05、0.79±0.02、0.30±0.01、0.93±0.01；Gli的mRNA相对表达量分别为1.00±0、1.35±0.04、0.25±0.02、0.65±0.04，蛋白相对表达量分别为0.63±0.01、0.96±0.01、0.57±0.01、1.10±0.01（图4）。提示硼替佐米作用于MM细胞系后，TFEB的下调逆转了硼替佐米对RPMI8226和U266细胞中Ptch1、Gli mRNA表达的抑制作用（*P*<0.05）（图3、4），Ptch1、Gli1的表达水平受TFEB调控。

**图3:**
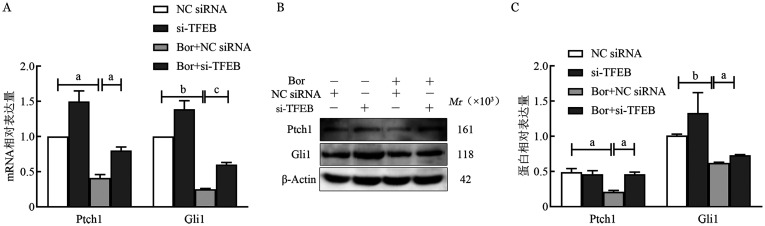
实时荧光定量PCR和Western blot法检测RPMI 8226细胞系转染转录因子EB（TFEB）后Ptch1、Gli1因子mRNA（A）和蛋白（B、C）相对表达量 **注** ^a^*P*<0.01，^b^*P*<0.001，^c^*P*<0.05；Bor：硼替佐米；NC siRNA：空载体；si-TFEB：小干扰RNA-TFEB

**图4:**
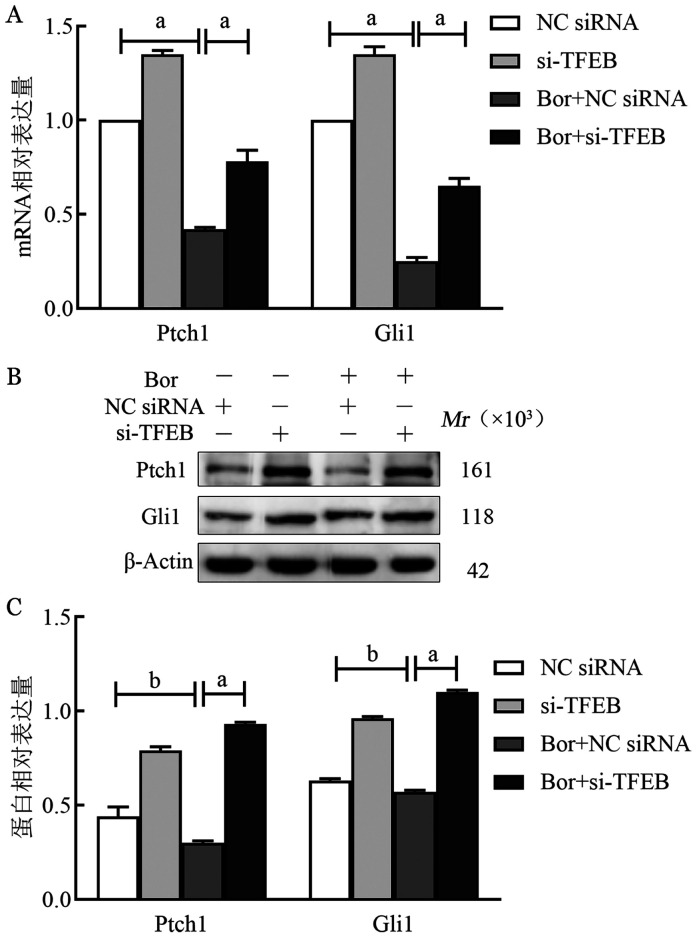
实时荧光定量PCR和Western blot法检测U266细胞系转染转录因子EB（TFEB）后Ptch1、Gli1因子mRNA（A）和蛋白（B、C）相对表达量 **注** ^a^*P*<0.001，^b^*P*<0.05；Bor：硼替佐米；NC siRNA：空载体；si-TFEB：小干扰RNA-TFEB

4. RT-qPCR和Western blot法鉴定Ptch1转染效率：提取OE-NC组、OE-Ptch1组细胞总RNA和总蛋白，RT-qPCR和Western blot法检测各组细胞中目的基因Ptch1的mRNA和蛋白相对表达量，结果显示，慢病毒转染OE-Ptch1后，在RPMI8226细胞系中，OE-NC组和OE-Ptch1组Ptch1因子mRNA相对表达量分别为1.00±0、16.99±4.95，Ptch1蛋白相对表达量分别为0.55±0.01、1.04±0.04（图5）。在U266细胞系中，OE-NC组、OE-Ptch1组Ptch1的mRNA相对表达量分别为1.00±0、8.26±2.54；蛋白相对表达量分别为0.34±0.01、0.96±0.06（图6）。OE-Ptch1组Ptch1的mRNA和蛋白相对表达量均明显高于OE-NC组（*P*值均<0.05）（图5、6）。

**图5:**
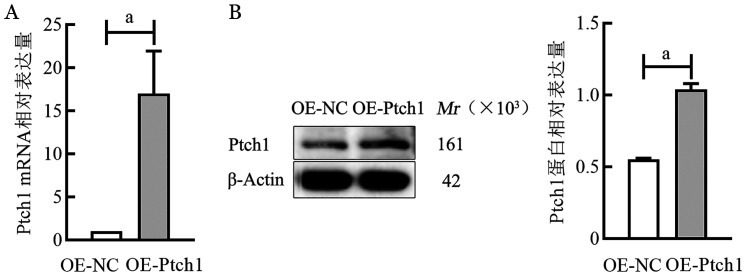
实时荧光定量PCR和Western blot法检测RPMI8226细胞系转染Ptch1后的Ptch1因子mRNA（A）和蛋白（B）相对表达量 **注** ^a^*P*<0.01；OE-NC：转染Ptch1对照质粒单克隆细胞；OE-Ptch1：转染Ptch1过表达质粒单克隆细胞

**图6:**
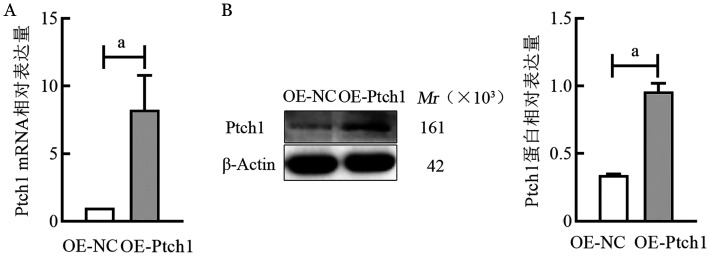
实时荧光定量PCR和Western blot法检测U266细胞系转染Ptch1后的Ptch1因子mRNA（A）和蛋白（B）相对表达量 **注** ^a^*P*<0.01；OE-NC：转染Ptch1对照质粒单克隆细胞；OE-Ptch1：转染Ptch1过表达质粒单克隆细胞

5. 吖啶橙染色及溶酶体探针观察自噬现象及溶酶体数量：吖啶橙染色分析显示，上调Ptch1可使硼替佐米作用后的两种MM细胞系自噬小体（棕黄色斑点）数量均减少（*P*值均为0.001）（图7A）；因Ptch1慢病毒转染带有绿色荧光，故用溶酶体红色探针检测各组细胞溶酶体数量，结果表明，上调Ptch1使硼替佐米作用后的两种MM细胞系溶酶体探针数量均减少（*P*值分别为0.001、0.007）（图7B、C）。

**图7:**
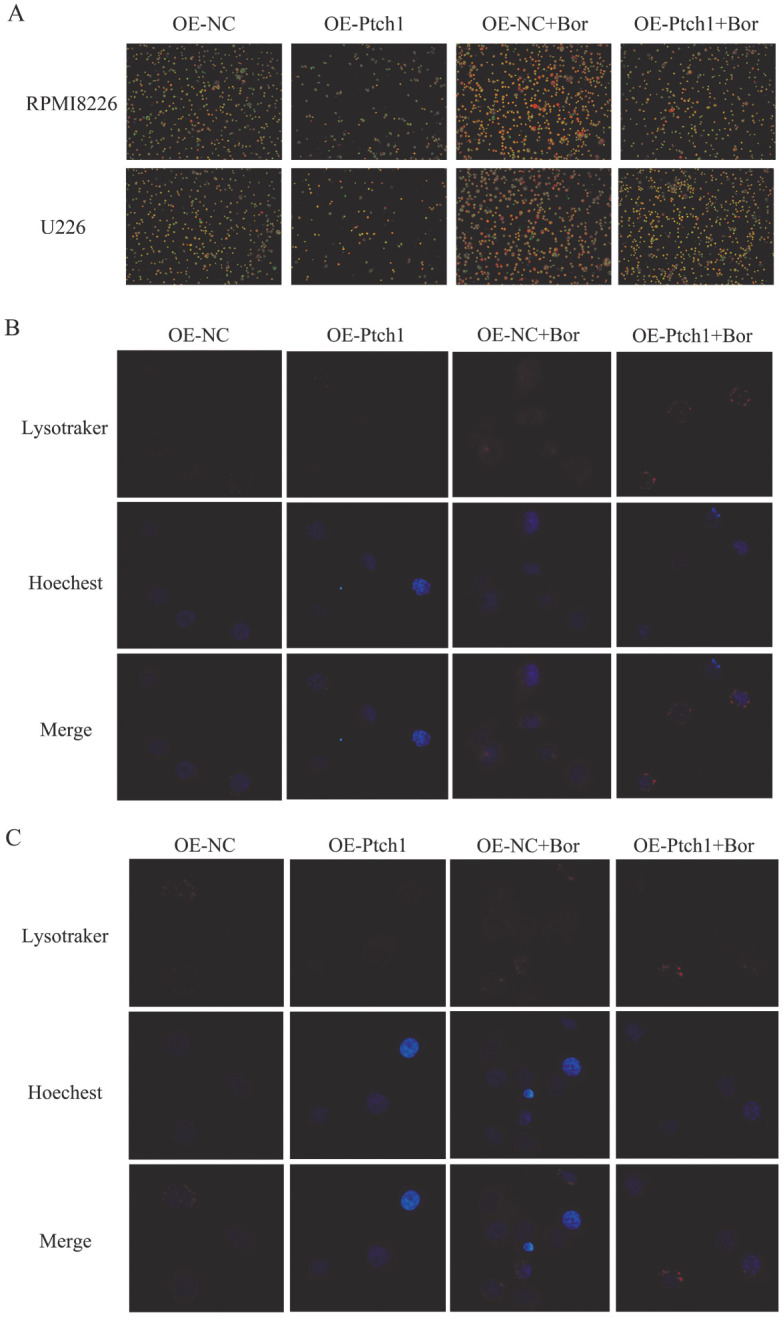
吖啶橙荧光染色（A）和溶酶体荧光探针（B、C）观察过表达Ptch1后RPMI8226细胞系（A、B）和U266细胞系（A、C）自噬囊泡及溶酶体数量变化 **注** Bor：硼替佐米；OE-NC：转染Ptch1对照质粒单克隆细胞；OE-Ptch1：转染Ptch1过表达质粒单克隆细胞

6. Western blot检测自噬溶酶体相关因子表达：结果表明，在RPMI8226细胞系中，OE-Ptch1组自噬相关因子LC3Ⅱ、Beclin-1、Lamp1的相对表达量（分别为0.07±0.01、0.60±0.01、0.23±0.01）明显低于OE-NC组（分别为0.66±0.01、0.93±0.02、0.51±0.01）（*P*值均<0.05）；Bor+OE-Ptch1组细胞自噬相关因子LC3Ⅱ、Beclin-1、Lamp1的表达水平（分别为0.75±0.02、0.75±0.02、0.42±0.01）较Bor+OE-NC组（分别为1.20±0.02、1.27±0.03、1.12±0.02）减少（*P*值均<0.05）。在U266细胞系中，OE-NC、OE-Ptch1、Bor+OE-NC、Bor+OE-Ptch1组自噬相关因子LC3Ⅱ蛋白相对表达量分别为0.61±0.04、0.15±0.01、1.38±0.11、0.49±0.03，Beclin-1蛋白相对表达量分别为0.71±0.04、0.35±0.01、1.41±0.06、0.78±0.41，Lamp1蛋白相对表达量分别为0.74±0.01、0.36±0.04、1.33±0.02、0.68±0.01，OE-Ptch1组自噬相关因子LC3Ⅱ、Beclin-1、Lamp1的表达较OE-NC组明显减少（*P*值均<0.05）；与Bor+OE-NC组相比，Bor+OE-Ptch1组细胞自噬相关因子LC3Ⅱ、Beclin-1、Lamp1的表达减少（*P*值均<0.05）（图8）。

**图8:**
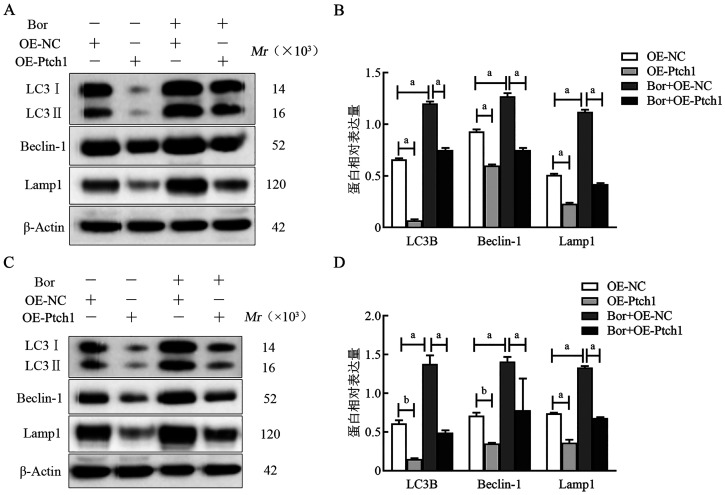
Western blot法检测过表达Ptch1后RPMI8226细胞系（A、B）和U266细胞系（C、D）自噬相关因子蛋白表达水平 **注** ^a^*P*<0.001，^b^*P*<0.01；Bor：硼替佐米；OE-NC：转染Ptch1对照质粒单克隆细胞；OE-Ptch1：转染Ptch1过表达质粒单克隆细胞

## 讨论

Hh信号通路是一种在生物体内发挥重要作用的配体依赖性信号传导通路，参与调控胚胎发育和组织器官形成过程，同时在多种组织干细胞的分化、增殖和凋亡中发挥调控作用[12]。该通路的活化始于Hh配体（如SHH、IHH、DHH）与跨膜受体帕奇蛋白（PTCH1、PTCH2）的结合，从而解除跨膜受体对SMO的抑制，进而激活下游转录因子家族（GLI1/2/3），调控靶基因（如Cyclin D1、MYC、BCL-2等）的表达[13]。在生理状态下，Hh通路的活性受到严格调控，然而，其异常激活与多种肿瘤的发生发展密切相关[14]。研究证明，MM的肿瘤微环境存在Hh通路的过度活化，可能通过促进肿瘤细胞增殖、耐药及骨髓基质细胞间的相互作用驱动疾病进展[15]。因此，深入探究Hh通路在MM中的分子机制或许能为靶向治疗提供新策略。

本研究收集硼替佐米作用后的MM细胞系，行全转录组基因测序，DNBSEQ平台检测Con组和Bor组表达有差异的mRNA，进一步应用GTRD网站分析TFEB的靶基因。以组间差异mRNA（4 153个）、TFEB的靶基因（1 620个）、Hh信号通路中相关因子（18个）为基础画韦恩图，结果显示，三者的共同因子为Ptch1。根据上述预测结果，Ptch1基因既是TFEB的靶基因，也是Hh的关键分子，故本研究拟筛选TFEB和Ptch1/Hh信号通路用于后续分子机制的深入研究。

“转录事件”是一个复杂回路的结果，涉及转录因子、转录激活因子或转录抑制因子、长链非编码RNA和miRNA的相互作用[16]–[18]。转录因子作为基因表达调控的关键因子，负责调节基因的转录过程，参与调控多个信号网络中基因的表达。增强子和抑制子分别对基因的表达起到增强和抑制的作用，通过调节这些基因的表达水平，转录因子影响多种疾病的发生和发展[19]。本研究通过GTRD网站分析Ptch1为TFEB的靶基因，将硼替佐米作用后的MM细胞系高表达的TFEB敲低，构建si-TFEB，将硼替佐米作用于转染si-TFEB后的MM细胞系，发现其对MM细胞系的增殖抑制作用较Bor+NC siRNA组减弱，敲低TFEB部分逆转了硼替佐米对MM细胞系的增殖抑制作用。同时RT-qPCR和Western blot法结果显示，TFEB的下调逆转了硼替佐米对MM细胞Ptch1、Gli1表达的抑制作用。这些数据表明Ptch1、Gli1的表达受到TFEB的负调控，相关内容目前还未见报道。

最新研究表明，在多种癌症中，Ptch1/Hh信号与自噬存在相互作用。Tang等[20]研究表明，下调Ptch1可诱导TGF-β1刺激的TCMK-1细胞自噬；在癌症中，Ptch1突变及功能缺失可能会刺激SW620结肠癌细胞自噬，促进对营养匮乏条件的适应，从而带来选择性优势[21]。此外，在肺癌的多个细胞系中也检测到了Hh信号的激活[22]，但是Ptch1/Hh信号通路与自噬的相关性在MM细胞系中未见报道。

前期实验验证了在硼替佐米作用的MM细胞系中，Ptch1/Hh的表达受到TFEB的调控。为进一步验证TFEB靶向Ptch1/Hedgehog信号通路诱导MM细胞自噬的假设，我们探讨了Ptch1对MM细胞系的自噬调节作用，将硼替佐米作用后的MM细胞系低表达的Ptch1过表达，结果发现过表达Ptch1部分逆转了硼替佐米对MM细胞系的增殖抑制作用，吖啶橙染色及溶酶体探针分析，上调Ptch1可使硼替佐米作用后的MM细胞系自噬囊泡及溶酶体数量减少，进一步Western blot分析显示硼替佐米作用于过表达Ptch1的细胞系中，其自噬相关因子LC3Ⅱ、Beclin-1、Lamp1较Bor+OE-NC组表达明显减少，上调Ptch1可逆转硼替佐米诱导的RPMI8226和U266细胞自噬。总之，本研究的实验结果显示TFEB可靶向Ptch1/Hh信号通路诱导MM细胞自噬，为更深入地了解硼替佐米治疗MM的机制提供了数据，为以后更好地应用硼替佐米提供了思路。
